# Information sharing between family and friend carers of older adults and healthcare professionals: Protocol for a systematic review of qualitative studies

**DOI:** 10.1371/journal.pone.0331717

**Published:** 2026-02-10

**Authors:** Steven Hall, Allison Cammer, Stephanie Chamberlain, Kelly Baskerville, Cavan Fani, Ziyu Li, Hannah M. O’Rourke

**Affiliations:** 1 Faculty of Nursing, University of Alberta, Edmonton, Alberta, Canada; 2 College of Pharmacy and Nutrition, University of Saskatchewan, Saskatoon, Saskatchewan, Canada; 3 Faculty of Nursing, University of Calgary, Calgary, Alberta, Canada; Public Library of Science, UNITED KINGDOM OF GREAT BRITAIN AND NORTHERN IRELAND

## Abstract

**Background:**

Population aging worldwide continues to intensify the demand for family and friend carers to support older adults. Informal carers have long identified information gaps as a key challenge in their caregiving role, underscoring the importance of effective communication and collaboration with healthcare professionals (HCPs).

**Objectives:**

To complete a comprehensive review of qualitative research and to synthesize what is known about the barriers and facilitators to information sharing between carers of older adults and HCPs.

**Methods:**

This protocol outlines a systematic review that follows the Preferred Reporting Items for Systematic Reviews and Meta-Analyses and PRISMA-P 2015 guidelines. We searched MEDLINE, EMBASE, CENTRAL, CINAHL, and PsycINFO on May 5, 2025. Our goal is to identify qualitative studies that meet our “PICo” (Population, phenomena of Interest, Context) eligibility criteria. Population includes carers and HCPs. Phenomena of interest is information sharing between the two populations. Context is carers who provide care to older adults aged 60 or above in any care setting. Two reviewers will independently screen titles and abstracts and full texts. We will use Covidence Extraction 2.0 for extraction of data from included records. Qualitative data from included records will be synthesized using Lockwood’s meta-aggregation and Sandelowski’s meta-summary approach to describe the experiences of information sharing reported within the literature.

**Discussion:**

By comprehensively evaluating barriers and facilitators to information sharing within the literature, this review will offer significant insights into supporting more effective communication between carers and HCPs. Findings will inform the development of new strategies designed to optimize communication processes between carers and HCPs, with the ultimate goal of improving outcomes for older adults requiring caregiving support. This synthesis of current evidence will also identify gaps for further investigation and underscore the need for innovative communication solutions in diverse healthcare contexts.

**Trial registration:**

PROSPERO CRD420250636906

## Background

Population aging is a global phenomenon driving the increasing need for family and friend (also known as “informal”) carers to support older adults [1]. As the number of older adults continues to grow, it is crucial to address the support needs of carers who play a vital role in providing support to the aging population [2,3]. Carers of older adults have consistently expressed the importance of information sharing between healthcare professionals (HCPs) and themselves about their care recipients, as highlighted in various studies [4–11]. Information sharing is essential for ensuring all parties involved in a patient’s care have access to accurate, timely, and comprehensive information [12]. A previous scoping review determined that the need for information about their care recipient was a support priority for carers [13]. As such, effective communication and sharing of information is crucial for ensuring high-quality patient care and outcomes.

### Rationale

Family and friend carers consistently describe information exchange with HCPs as a hallmark of safe, person-centred care for older adults, yet the evidence base remains fragmented. The most closely related synthesis to our knowledge is an integrative review by Morrow and Nicholson [14]. In their review, they observed information sharing as only one of six components of engagement. Their review was restricted to acute care settings and was published 10 years ago. To our knowledge, no systematic review using qualitative synthesis methods has since addressed this gap.

However, recent qualitative studies have highlighted the topic and the heterogeneity of contexts in which problems persist. For example, carers of head and neck cancer patients have reported inadequate communication about post-surgical side-effects and difficulty accessing tailored education before discharge [15]. In a study by Ferguson et al. [10], heart failure caregiver-patient dyads and HCPs described unmet informational needs regarding symptom recognition, medication management, and navigation of health services. Furthermore, in a study by Leone et al. [16] describing rural care settings, family and friend carers of persons with dementia explained the experience of having to search on their own for trustworthy service information with limited guidance from HCPs. Lastly, in Chinese nursing homes, lack of honest information sharing and low trust were major barriers to engaging residents and family and friend carers in advance care planning conversations [17]. Knowledge exists on information sharing within the wider literature and this planned systematic review aims to consolidate the existing knowledge of the concept of information sharing between carers and HCPs.

### Objectives

The objective of this planned systematic review of qualitative studies is to synthesize what is known about the barriers and facilitators to information sharing between carers of older adults and HCPs. The two overarching research questions guiding this systematic review are:

1. What are the reported experiences of information sharing between family and friend carers of older adults and HCPs?
2. What barriers and facilitators influence information sharing in these interactions across care settings?

## Methods

To achieve the aforementioned objectives, we are using the systematic review approach with meta-aggregation [18] and meta-summary [19] as suggested by the “Right Review” tool [20] and in consultation with a health sciences librarian. Our systematic review will be guided by the Cochrane Handbook for Systematic Reviews [21] and reported in accordance with the Preferred Reporting Items for Systematic Reviews and Meta-Analyses [22] statement [22]. This protocol is structured in accordance with PRISMA-P 2015 guidelines [23,24]. The PRISMA-P 2015 checklist is included as S1 File. The review team is led by a masters-prepared registered nurse and PhD-trainee (SH). The team currently comprises of a PhD-prepared registered nurse (HMO); a PhD-prepared registered dietitian (AC); a PhD-prepared health services researcher (SC); a masters-prepared registered nurse research associate (KB); and two undergraduate nursing (BScN) honours students (CF and ZL). Given the topic’s relevance to health service delivery, this team is well positioned to take on this review.

### Registration and current status

Our protocol is registered in the Prospective Register of Systematic Reviews (PROSPERO) database (registration number: CRD420250636906). We carried out our search on May 5, 2025, and conducted a preliminary review of records on Covidence [25]. As of January 20, 2026, we continue to screen titles and abstracts of 5448 records that were located from our information sources, mentioned later in this protocol.

### Eligibility criteria

As recommended in the literature on qualitative meta-aggregation [18], we used the “PICo” mnemonic (Population, phenomena of Interest, Context) to craft our research question and set our eligibility criteria. Eligibility criteria for inclusion in this planned systematic review will be (1) a sample population of family and friend carers or HCPs; (2) a phenomena of interest being information sharing between carers and HCPs; and (3) the context is care of older adults in any setting.

There will be no date limits imposed on inclusion to capture the breadth of literature on this topic and to examine if any changes in needs or priorities for information sharing have occurred over time. In addition to the PICo variables, inclusion criteria will be: completed research studies that are qualitative or mixed methods with a relevant qualitative component. Findings in the qualitative studies or qualitative components of mixed methods studies must report participants’ experiences of information sharing. Exclusion criteria will include: editorials, clinical case studies, reviews, expert opinion papers, and studies that were published as abstracts only. Considering the review team is fluent in English only, we will reach out to other faculty members in our department for assistance with translating records not published in English. If a record is published in a language other than English of which we cannot find a colleague to translate, we will use DeepL [26] software, a reliable translation tool that was recommended during our consultation with the health sciences librarian. Eligibility criteria are also listed in S2 File and are described in more detail using the PICo framework in the following paragraphs.

#### Population.

The population of focus is family and friend carers (also referred to as informal carers or caregivers) interacting with HCPs, or HCPs interacting with family and friend carers. Family and friend carers are defined as people who take on an “unpaid caring role for someone who needs help because of a physical or cognitive condition, an injury or a chronic life-limiting illness” [27]). The operational definition of caregiving to be used in this review was crafted in a concept analysis, comprehensive of nursing, sociology, and psychology perspectives:

“Caregiving is the process of helping another person who is unable to do for themselves in a ‘holistic’ (physically, mentally, emotionally, and socially) manner. Caregiving is facilitated by certain character traits, emotional skills, knowledge, time, and an emotional connection with the care recipient” [28, p. 15].

#### Phenomena of interest.

The phenomena of interest to be examined in this systematic review is “information sharing” between family and friend carers and HCPs, involving the exchange of relevant patient health information to support coordinated, safe, and effective care [12,17] . An included study can examine either population’s experience (i.e., HCPs information sharing experiences with carers or carers information sharing experiences with HCPs). Information sharing involves the transfer or exchange of patient-related data, such as medical history, care plans, test results, and follow-up needs, between caregivers and HCPs to facilitate decision-making and continuity of care [12,29]. It can be bidirectional, with both caregivers and HCPs contributing and accessing information, and may occur through various means, including written documents, electronic health records (EHRs), and direct communication [30,31]. For the purposes of this review, we will focus on information sharing via direct communication.

#### Context.

Carers and HCPs must be caring for older adults within the study. Due to our qualitative synthesis approach and the previously iterated review objectives, any care delivery setting is appropriate for inclusion. The World Health Organization [1] defines “older adults” as 60 years or older, hence our decision to set the age threshold for care recipients at >60 years. Studies will be included if the mean study population age is care recipients who are 60 years or above or if the study includes a subgroup analysis of carers to these individuals. If the study does not report the age of care recipients, the study will be included if it is narratively indicated that the carers are caregiving to older adults.

### Information sources

When undertaking a systematic review, the Cochrane Handbook for Systematic Reviews cites MEDLINE, EMBASE, and CENTRAL as essential databases to search [21]. As such, we conducted a search of these three databases, along with the Cumulative Index to Nursing and Allied Health Literature (CINAHL) and PsycINFO, which are specialized databases with a related focus to the topic of this review. These five databases were accessed using the University of Alberta’s institutional license on May 5, 2026. MEDLINE, EMBASE, and PsycINFO were accessed using Ovid interface; CINAHL was accessed using EbscoHOST; and CENTRAL was accessed using the Cochrane Library interface.

### Search

The search strategy for this systematic review was developed by the first author (SH) in consultation with a health sciences librarian at the University of Saskatchewan’s Leslie and Irene Dube Health Sciences Library (EL). Filters to retrieve studies related to the topic of this review were retrieved from the University of Alberta Library’s Health Sciences Search Filters collection [32]. This included the pre-prepared search filters for the older adult population, health professionals, and allied health professionals. Search filters for carers were adapted from a previous scoping review’s search strategy [13]. Search terms for communication or information sharing were initially identified using ChatGPT-4o [33] by querying “Create a single line Ovid search filter for communication or information sharing.” MeSH terms and other synonyms were then identified manually and added to the search line. The final search strategy was reviewed and approved by the health sciences librarian. A sample search strategy for MEDLINE (Ovid interface) is presented in Table 1. Our search strategy was conducted across the five databases on May 5, 2025. Search strategies for all five databases are provided in S3 File.

**Table 1:** Search Strategy run on May 5, 2025, using Ovid MEDLINE(R) Epub Ahead of Print and In-Process, In-Data-Review & Other Non-Indexed Citations and Daily.

#	Searches	Results
1	exp Geriatrics/ or exp Aged/ or Health Services for the Aged/ or Senior Centers/ or (elders or elderly or geriatric* or gerontolog* or “old age” or “senior citizen*” or (seniors not “high school”) or ((older or mature) adj3 (adult* or person* or people or patient or patients or man or men or woman or women)) or centenarian* or nonagenarian* or octogenarian* or septuagenarian* or sexagenarian* or dottering or decrepit or tottering or overaged or “oldest old” or supercentenarian*).mp.	3940510
2	exp Caregivers/ or (Carer* or caretak* or care tak* or care-tak* or caregiv* or care-giv*OR care giv* or carepartner* or care-partner* or care partner*).mp.	152451
3	exp Health Personnel/ or (acupuncturist* or allergist* or anatomist* or an?esthesiologist* or anesthetist* or audiologist* or cardiologist* or chiropractor* or clinician* or dentist* or dermatologist* or diabetologist* or dietician* or doctor* or doula or doulas or endocrinologist* or gastroenterologist* or general practitioner* or geriatrician* or gynecologist* or h?ematologist* or ((health or healthcare or hospital or medical) adj2 (worker* or workforce or personnel or practitioner* or provider* or professional* or employee* or staff* or navigator*)) or hospitalist* or immunologist* or intensivist* or internist* or medical resident* or midwife or midwives or neonatologist* or nephrologist* or neurologist* or neurosurgeon* or nurse or nurses or nutritionist* or obstetrician* or oncologist* or ophthalmologist* or optometrist* or osteopath or osteopaths or otolaryngologist* or pathologist* or p?ediatrician* or pharmacist* or pharmacologist* or phlebotomist* or physician* or podiatrist* or prosthetist* or psychologist* or psychiatrist* or pulmonologist* or radiographer* or radiologist* or radiotherapist* or rheumatologist* or surgeon* or therapist* or toxicologist* or urologist* or veterinarian*).mp.	2791924
4	exp Allied Health Personnel/ or Doulas/ or (allied health profession* or allied health* personnel or allied health* staff or allied health* practitioner* or allied health* employee* or allied health* worker* or athletic trainer* or audiologist* or community health worker* or counsellor* or counselor* or cytogenetic technologist* or dental auxiliaries or dental auxiliary or dental assistant* or dental hygienist* or dental technician* or denturist* or diagnostic molecular scientist* or dietitian* or doula or doulas or emergency medical technician* or EMT or EMTs or exercise physiologist* or family therapist* or health educator* or health information technologist* or healthcare assistant* or healthcare support worker* or histotechnologist* or home health* aide* or kinesiologist* or kinesiotherapist* or lactation consultant* or licensed practical nurse* or LPN or LPNs or medical dosimetrist* or medical laboratory scientist* or medical physicist* or medical records administrator* or medical receptionist* or medical secretar* or music therapist* or midwife* or nurse* aide* or nuclear medicine technologist* or nutritionist* or occupational therapist* or operating room technician* or ophthalmic assistant* or paramedic* or pathologist* assistant* or p?ediatric assistant* or pharmacy technician* or physical therapist* or physical therapy assistant* or physician assistant* or physiotherapist* or population program specialist* or prosthetist* or prosthet* technician* or psychiatric aide* or radiation therapist* or radiographer* or respiratory therapist* or sonographer* or (speech adj2 pathologist*)).mp.	228121
5	exp Health Communication/ or exp Health Education/ or (information-sharing or information sharing or information-exchange or “information exchange” or “knowledge transfer” or “data sharing” or “health communication” or “patient education” or “interpersonal communication”).mp.	326220
6	1 and 2	42247
7	3 or 4	2867482
8	5 and 6 and 7	1689

### Study records

#### Data management.

Full citations of records were exported from the five databases as.RIS files and uploaded to Covidence review management software [25] for title and abstract screening and subsequent full text screening. Duplicates were removed using automation tools in Covidence, which has shown to have 99.6–100% specificity [34]. In total, there were 5448 records to be screened. Title and abstract screening are being conducted in alphabetical order (records sorted by title, instead of Covidence’s default “machine-learned relevance”) to manually identify any further duplicates. Subsequently, full texts of records that pass the title and abstract screening phase will be sought from the University of Alberta’s library databases. After full text screening, studies that are included will undergo extraction using Covidence Extraction 2.0, a tool which is “best suited for non-intervention reviews with a customizable structure” [38].

#### Selection process.

Titles and abstracts of studies retrieved using the aforementioned search strategy are currently undergoing dual screening (two people screening each record). Dual screening is recommended over a single-screening process, as up to 13% of studies can be missed with only one screener [35]. Before initiating the screening process, screeners KB, CF, and ZL were invited to individual Covidence files that contained the first 50 records from the MEDLINE database. SH screened these 50 titles and abstracts with KB, CF, and ZL to ensure there was an understanding of the screening criteria. Our Cohen’s Kappa values were 0.2674, 0.5136, and 0.1131 respectively, which indicates an initial low agreement [36]. However, the actual total number of conflicts was between 6 and 8, and the opportunity for a higher Kappa was limited because this was a pilot process of only 50 studies. Due to this pilot screening process, we were able to discuss and scrutinize areas for this low agreement, thus supporting our screening process to have higher inter-rater reliability going forward.

Now, using Covidence, all titles and abstracts are being screened by the lead author (SH) and one of the other screeners (KB, CF, or ZL). Conflicts that arise at the title and abstract screening phase will be resolved through discussions. Conflicts that cannot be definitively resolved in discussion between screeners will be resolved by the senior supervising author (HMO). Subsequently, all full text papers will be screened by SH and another screener. Any conflicts arising at this stage over the eligibility of studies will be resolved in the same manner as previously described. In instances where multiple articles report similar results from the same data set, we will use the part of the PRISMA diagram template where you can identify that there are additional reports coming from “X” studies (e.g., Studies included in review: n = 15; Reports of included studies: n = 16). We will also use CitationChaser online software [37] to screen reference lists and citing articles of studies that are included after the first complete round of title/abstract and full text screening. Instructions for the screening process and inclusion forms for the full texts are provided in S2 File.

### Data collection process

We will use the Covidence Extraction 2.0 template [25] for data extraction. We will pilot this template with team members participating in the extraction process. Each participating team member will extract data from three studies as a test of understanding, to be subsequently reviewed by the lead author (SH). Once understanding of the extraction process is confirmed, the team will divide the included records to extract data. SH will review extraction conducted by other team members. Data will be extracted from included articles by one team member independently. All extracted items will be reviewed and extraction items within exported.CSV tables will be “cleaned” by SH to confirm accuracy. We will contact corresponding authors of included articles for any missing details or extraction items not reported within the original article.

### Data items

Data items to be extracted from included records include general and administrative information; characteristics of both the carer participants and their care recipients; characteristics of healthcare professional participants; study characteristics; and key study findings (e.g., themes or categories of qualitative data). Rationale for each data item and tentative instructions for extraction are presented in Table 2. Furthermore, entire Findings sections from included articles will be extracted into separate documents and imported into NVivo 14 [38] for qualitative data analysis.

**Table 2:** Data items, rationales, and instructions for extraction.

Data Item	Rationale	Instructions	Example Note: If *not reported*, record in table as “N/R.” If *not applicable*, record in table as “N/A.”
***General and Administrative Information***			
Title	Record keeping, organization, and identification.	Copy the title verbatim into the extraction form.	This is a sample title of an article: Copy it into the table verbatim
Author, Year	Record keeping, organization, and identification.	Record the first author’s last name. Include a second author’s last name if only two authors or use “et al.” for three or more authors. Record year of publication following the author’s name.	Hall, 2025 OR Hall & O’Rourke, 2025 OR Hall et al., 2025
Place of Study	The geographic location of a study influences cultural factors, healthcare policy, and economic factors relevant to information sharing as a phenomenon of interest. Understanding the setting helps contextualize findings and assess generalizability.	Record the name of the country where the study took place. If more than one country, list each country and separate using semicolons.	Canada OR Canada; United Kingdom
***Participant Characteristics***			
Carer Sample Size	Number of carer participants indicates the study’s size and scale. If the study was HCPs discussing their communication with carers, this can be omitted with appropriate documentation.	Record total N of carers in study.	50
Carer Age	Age may influence the type of caregiving challenges.	Record the mean age of carers reported.	60.1
% Women	Caregiving is often performed disproportionately by women, so extracting gender distribution can highlight gender-related trends or potential disparities. This detail also informs equity considerations.	Record the percentage of carers identifying as women reported for both intervention and comparison group. Separate with a semicolon.	45%
Carer-Care Recipient Relationship	Whether the carer is a spouse, adult child, or friend can influence caregiving responsibilities and needs. This helps identify relational contexts.	Record the carer’s relationship to the care recipient as a percentage for both intervention and comparison group. Separate relationships with a semicolon.	Spouse = 58%; Adult child = 38%; Friend = 4%
Carer Education Level	Education level may influence carers’ ability to understand medical information and navigate health systems.	Record the n of carers per education level as a percentage. Separate education levels with a semicolon.	High School = 20%; College Diploma = 40%; University Degree = 40%
Carer Ethnicity	Cultural background may shape communication preferences, health beliefs, and caregiving norms. Capturing ethnicity helps evaluate cultural relevance.	Record the n of carers per ethnicity as a percentage. Separate ethnicities with a semicolon.	White = 30%; Black = 50%; Asian = 10%; Hispanic = 10%.
Carer Sexual Orientation	Sexual and gender minority carers may face unique barriers or experiences in healthcare settings. Documenting this allows for an understanding of inclusivity across diverse groups.	Record the n of carers per sexual orientation as a percentage for both. Separate sexual orientations with a semicolon.	Heterosexual = 20%; Lesbian = 30%; Gay men = 30%; Transgender = 20%.
Carer Employment Status	Employed carers may have distinct scheduling needs or higher stress levels related to balancing caregiving and work responsibilities. This information helps in assessing how working carers are accommodated or impacted.	Record the n of carers per employment status as a percentage. Separate employment statuses with semicolon.	Employed = 40%; Unemployed = 60%.
Average Length of Time as a Carer	New carers may have different informational needs compared to those who have been caregiving for more years.	Record the average length of time in caregiving role in years as a single number. If ranges are reported, copy ranges into table. Separate ranges with semicolons.	6 OR 6m-1y = 10%; 1-5y = 30%; > 5y = 70%.
Support from Others	Carers who have additional support (e.g., from family or community services) may have different experiences than those who are caregiving independently.	Record as Y or N in percentages. Separate Y and N in table with semicolon.	Y = 8%; N = 92%.
Average Hours per Week Engaging in Caregiving Activities	The intensity of caregiving (hours/week) reflects the potential burden. Higher caregiving hours may amplify stress and burden and impact information needs.	Record the average hours per week as a single number. If ranges are reported, copy ranges into as a percentage. Separate ranges with semicolons.	40 OR 0-20h = 20%; 20-40h = 20%; > 40h = 60%.
Care Recipient Sample Size	Some studies may involve multiple care recipients per carer. It also aids in understanding the representativeness of each study’s sample.	Record the total number of care recipients reported.	52
Care Recipient Age	Confirms the population of interest (older adults). As well, the older the care recipients are, they may present different caregiving and communication needs.	Record the mean age of care recipients reported.	80.3
Care Recipient Medical Condition	Many studies examine specific conditions (e.g., dementia, cancer). Documenting the primary health issues of older adults clarifies the context and potential transferability.	Record the care recipient’s medical condition as a percentage Separate conditions with a semicolon.	Dementia = 50%; Cancer = 50%.
Healthcare Professional Sample Size	Number of HCP participants indicates the study’s size and scale. This is applicable if the study includes or focuses on HCPs in their sample for data collection. They can be the primary source of data as well, if the study focuses on HCP experiences information sharing with carers.	Record the total number of HCPs in the study. If multiple HCP groups are participants within the study, list the overall total.	22
Healthcare Professional Profession	Different professional groups (e.g., nurses, physicians, pharmacists) have varying roles in information sharing. Recording the disciplines involved helps understand collaboration contextually.	Record the types of HCPs participating (e.g., nurses, physicians), along with their proportions if reported. Separate each profession with a semicolon. If only one group is specified, just name it.	Nurses = 40%; Physicians = 35%; Social Workers = 25% OR Nurses
Healthcare Professional Mean Years of Practice	Level of experience may influence how HCPs communicate with carers. This detail provides insight into whether professional experience moderates the experience of information sharing.	Extract the average (mean) years of professional experience for HCP participants as a single number, if available.	10.5
***Study Characteristics***			
Design	The study design (e.g., qualitative, mixed methods) informs the type of evidence.	Identify and record the study design (e.g., qualitative, mixed methods). If additional design details are reported (e.g., phenomenology, grounded theory, etc.), include them following a semicolon.	Qualitative OR Qualitative; grounded theory
Eligibility Criteria	Knowing which participants were eligible clarifies to whom the study results are most generalizable. It also helps evaluate consistency across included studies.	Summarize the inclusion and exclusion criteria that determined which participants were enrolled. Use semicolons to separate key points.	Inclusion: Age ≥ 60; unpaid informal caregivers providing ≥8 hours/week. Exclusion: Professional caregivers; palliative care only.
***Study Results***			
Key Findings	The reported key findings from a study will help to aggregate and summarize the main outcomes of qualitative and mixed methods studies that examine information sharing between carers and HCPs. In mixed methods studies, we are only extracting qualitative data.	List the key findings identified. Separate each theme with a semicolon. **Note:** The entire findings sections of all included studies will also be analyzed separately using NVivo [38].	Carers require more structured conversations when receiving information from HCPs; HCPs require more training on how to communicate with carers.
Qualitative Themes or Categories	Many qualitative studies report findings in themes or categories. Recording the names of these will allow for comparison in our meta-aggregation and meta-summary.	List the main themes or categories identified. Separate each theme with a semicolon.	Emotional Strain; Need for Flexible Communication; Trust in Clinical Team
***Miscellaneous Data***			
Miscellaneous Extractor Notes	Allowing space for open-ended notes ensures that any unexpected details, clarifications, or reviewer reflections can be documented.	Use this field for any additional details or clarifications not captured elsewhere. Include relevant quotes or clarifications on ambiguous data. If no extra notes, leave blank.	Study authors mentioned a high turnover of staff.

### Risk of bias in individual studies

For included records, we will use the JBI Critical Appraisal Checklist for Qualitative Research [39], which is recommended for use in qualitative systematic reviews [18]. Two authors (SH and KB/CF/ZL) will complete a critical appraisal using the JBI checklist and we will engage in discussions to ensure consensus is met. Our critical appraisal results will be reported narratively and provided in a Supplementary File with the completed review.

### Synthesis

This review will use qualitative meta-aggregation and meta-summary to synthesize the included studies’ findings. Meta-aggregation is a structured method aligned with conventional systematic review standards, where the reviewer avoids re-interpretation of the original qualitative data and instead collates findings as reported by the primary authors [18]. In this approach, commonalities and differences across studies are identified, and primary study findings are first grouped into thematic categories, then further distilled into synthesized conclusions that directly address the review question [18]. Qualitative meta-summary is a complementary technique to add a quantitative dimension to qualitative synthesis [32]. It involves the systematic extraction, grouping, and formatting of qualitative findings, followed by calculating frequency and intensity effect sizes to gauge the prevalence of each finding across the included studies [19]. By integrating these two methods, the synthesis will produce a narrative of themes (meta-aggregation) and a quantitative indication of how widely each theme is supported across studies (meta-summary). All extracted qualitative findings will be imported into NVivo 14 [38] to facilitate the analysis. NVivo will be used to manage the data, support coding of text, and organize findings by source. Using NVivo’s functions, we will be able to efficiently group similar concepts and also track the number of sources (studies) associated with each code or category, which will aid in computing effect sizes for the meta-summary component.

#### Meta-aggregation.

Using NVivo, SH (in collaboration with the team) will qualitatively code the extracted findings using the method of content analysis [40]. Each discrete finding or meaningful unit of text will be assigned one or more descriptive codes that capture its essence [18]. This coding is primarily inductive and data driven [40]. Codes will reflect concepts or issues reported in the information sharing experiences of carers and HCPs (e.g., “communication barriers” or “trust building”). Similar or duplicate findings from different studies will be given the same code. After open coding, the resulting codes will be examined for patterns and overlap. Codes that share a similar meaning or address a common facet of information sharing will be grouped into higher-order categories. Once the categories are finalized, we will synthesize them to generate overarching review findings. This involves translating each category into a coherent synthesized finding as a narrative statement that conveys the combined insight of all findings in that category. The synthesis will aggregate the evidence. Multiple similar findings will be condensed into a smaller number of higher-level conclusions. This progressive reduction (from many findings to a moderate number of categories, to a concise set of synthesized findings) is characteristic of meta-aggregation [18].

#### Meta-summary.

The meta-summary approach will allow us to quantify the prominence of each theme using two types of effect sizes for the findings: frequency effect size and intensity effect size [19]. Effect size calculations will be facilitated by the querying functions in NVivo 14 [38] by obtaining the number of sources coded at each node (for frequency) and the number of coded references per source (for intensity). Considering this systematic review is a qualitative synthesis, this quantitative supplementation does not denote statistical significance, but it helps convey how robust or widespread each finding is across the dataset.

**Frequency effect size.** For each thematic category, we will determine how many of the included studies reported that finding. The frequency effect size is calculated as the percentage of studies that contain a given finding [19]. For example, if 4 out of 20 studies in our review describe a particular issue in information sharing, the frequency effect size for that issue would be 20%. A higher frequency effect size indicates that a theme is more widely reported across the evidence base, suggesting it is a more prevalent or common experience [19]. Conversely, a low frequency (e.g., a theme appearing in only one or two studies) may point to a more unique or context-specific finding [19]. Following recommendations by Sandelowski et al. [41], we may use a cut-off (such as > 10% or > 20% frequency) to highlight particularly common findings, ensuring that our discussion focuses on the most salient themes while still noting less frequent insights for comprehensiveness.

**Intensity effect size.** We will also compute intensity effect sizes to understand each included study’s contribution to the overall breadth of findings. The intensity effect size for a given study is the percentage of all extracted findings that came from that study [19]. For instance, if a particular study contributed 5 out of a total of 20 distinct findings identified across all studies, that study’s intensity effect size would be about 25%. This measure helps identify if some studies are especially rich in relevant data (contributing many findings) or if the findings are more evenly distributed [19]. The intensity effect provides context on the weight of evidence each study carries in the synthesis [42].

### Review timeline

We ran our search strategy on May 5, 2025, and title and abstract screening of 5448 records in Covidence is approximately 30% complete as of January 20, 2026, with 46 full texts currently waiting to be screened at the next stage. Based on this measure, we are projecting approximately 150 full texts to review after title and abstract screening is complete. We expect title and abstract screening to be completed by January 30, 2026. Subsequently, we expect full text screening and extraction to be completed by March 30, 2026. Our analysis and final report are planned to be completed by May 31, 2026.

## Discussion

We anticipate that the results of this systematic review will inform future research efforts by identifying barriers and facilitators to information sharing between carers of older adults and the HCPs. Findings from this systematic review will also help to inform the development of future studies to co-design information sharing interventions for older adults and their carers. The knowledge translation strategy for this review will include presenting an abstract for the review at national and international conferences focusing on the care and wellbeing of older adults. Translating the knowledge collated from this review will draw attention to the information needs of family and friend carers, who are essential to the healthcare landscape in the context of an aging population. Ultimately, this systematic review is an important step toward improving carer engagement, satisfaction, and preparedness, thereby contributing to more patient-centered, efficient, and effective care for older adults.

## Supporting information

S1 File PRISMA-P 2015 Checklist. (DOCX)

S2 File Title & Abstract Screening Instructions. (PDF)

S3 File Search Strategies from All Databases Searched. (DOCX)
