# Supplementary material for: Information sharing between family and friend carers of older adults and healthcare professionals: Protocol for a systematic review of qualitative studies
Source: PLoS One. 2026 Feb 10;21(2):e0331717. doi: 10.1371/journal.pone.0331717 (PMC12890094; doi:10.1371/journal.pone.0331717)
Supplement: S2 File — (PDF) [file pone.0331717.s002.pdf]

Supplementary File 2

Title & Abstract Screening Instructions

1. Access screening via https://Covidence.org/. SH will provide access to the Covidence screening file.

2. Select "Continue" on screening for titles and abstracts. Example below:

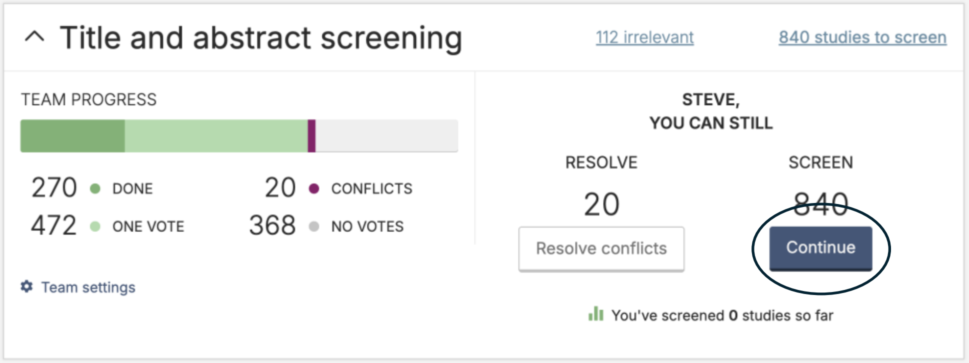

3. Follow the inclusion / exclusion criteria on the next page, which is also available on Covidence under "Criteria."

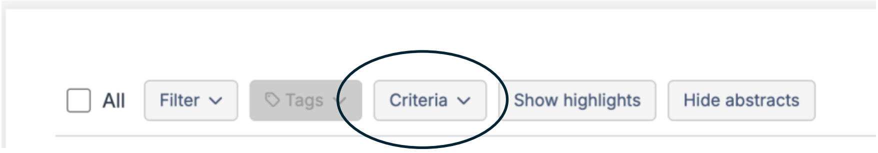

4. Select either "Yes" or "No" to log your decision. Do NOT use "Maybe" – use "Yes" instead and we will review as a team later if a conflict is created.

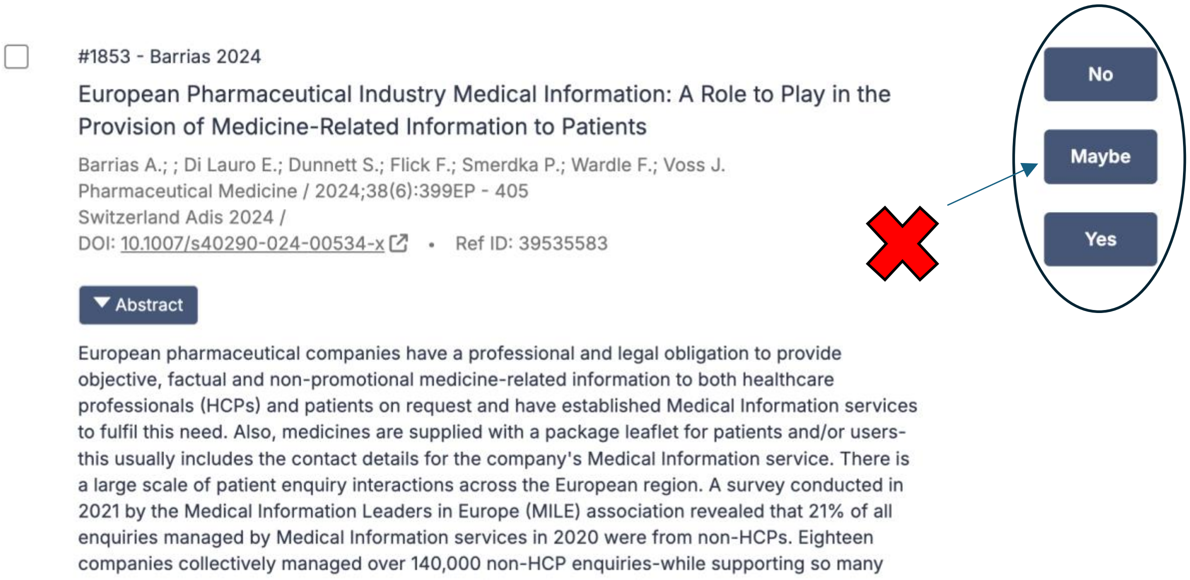

Inclusion / Exclusion Criteria

**Inclusion Criteria**	**Exclusion Criteria**
Study Characteristics	
• Complete reports of qualitative and mixed methods studies	• Purely quantitative work, editorials, case studies, reviews, expert opinion papers, studies published as abstracts only, and incomplete work.
Population	
• Family and friend carers and/or healthcare professionals	• Family and friend carers and/or healthcare professionals
Phenomenon of Interest	
• Information sharing experiences between carers and HCPs.<br>• Can be delivered in any setting (e.g., acute care, community or primary care, long term care, etc.).<br>• Can examine either population's experience (i.e., HCPs information sharing experiences with carers or carers information sharing experiences with HCPs).	• Does not describe the experience of information sharing.<br>• Information sharing with the care recipient, rather than the carer.
Context	
• Care recipients are older adults aged 60 and above. Can be noted narratively (e.g., states population is carers of older adults in title or abstract) and/or by mean age.	• Care recipient population is not older adults or is <60 years in mean age.

Note: If unsure about a study based on the abstract, vote "Yes" for the team to either explore the full text later or resolve during Conflict Resolution.

Full Text Screening Instructions

1. Full text PDFs will be available on Covidence following completion of Title & Abstract screening. Click the link under "Full text" to access. Example below:

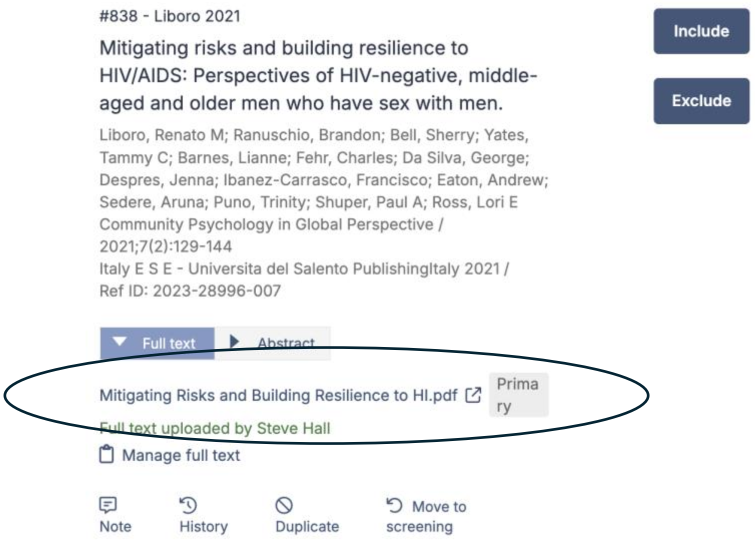

2. If excluding the text, please rank the primary reason for exclusion according to the rank order listed below. An example of how to select the reason is shown below this list.
    a. Full text not available
    b. Wrong language
    c. Wrong population
    d. Wrong design
    e. Wrong intervention
    f. Wrong comparator
    g. Wrong outcome
    h. Wrong outcome measure

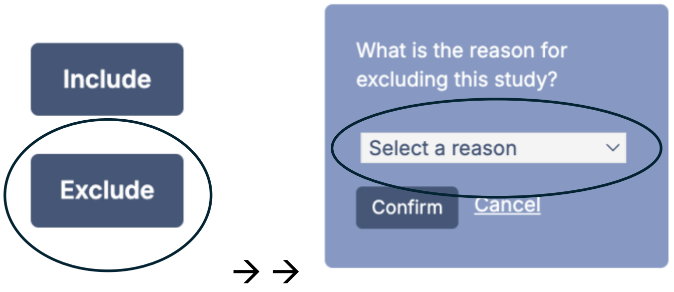

Select "Exclude" then choose reason from drop-down box.

3. If including the text, use the inclusion screening form on the following page for documentation.

Full Text Inclusion Screening Form

Reviewer Name: ________________________ Date of Review: ________________________

First Author of Study, Year: ____
(e.g., Hall, 2025)

First 2 Words in Title: ____
(e.g., Exploring information…)

**REVIEW FULL ARTICLE:**	Yes	No	Don't Know
Design: Is the study collecting qualitative data? (i.e., qualitative or mixed methods study)	☐	☐	☐
Language: Is the study published in English or able to be translated via DeepL? Test this by translating the study's abstract and examining the translated text for coherence.	☐	☐	☐
Population and Context: Is the population carers and/or healthcare professionals who work with carers?<br>Please indicate which: Carers ☐ Healthcare Professionals ☐	☐	☐	☐
Phenomena of Interest: Does the study report on information sharing between carers and HCPs?	☐	☐	☐
Context: Are the carers and/or HCPs providing care to older adults (>60 years old)?<br>If yes, is it identified narratively ☐ and/or by mean age ☐	☐	☐	☐

Adapted from Hempel S, Shetty KD, Shekelle PG, et al. Machine Learning Methods in Systematic Reviews: Identifying Quality Improvement Intervention Evaluations [Internet]. Rockville (MD): Agency for Healthcare Research and Quality (US); 2012 Sep. Appendix C, Inclusion Screening Form for Publications Obtained as Full Text. Available from: https://www.ncbi.nlm.nih.gov/books/NBK109710/
