# Supplementary material for: Information sharing between family and friend carers of older adults and healthcare professionals: Protocol for a systematic review of qualitative studies
Source: PLoS One. 2026 Feb 10;21(2):e0331717. doi: 10.1371/journal.pone.0331717 (PMC12890094; doi:10.1371/journal.pone.0331717)
Supplement: S3 File — (DOCX) [file pone.0331717.s003.docx]

**Ovid MEDLINE(R) Epub Ahead of Print and In-Process, In-Data-Review & Other Non-Indexed Citations and Daily <May 02, 2025>**

1 exp Geriatrics/ or exp Aged/ or Health Services for the Aged/ or Senior Centers/ or (elders or elderly or geriatric* or gerontolog* or "old age" or "senior citizen*" or (seniors not "high school") or ((older or mature) adj3 (adult* or person* or people or patient or patients or man or men or woman or women)) or centenarian* or nonagenarian* or octogenarian* or septuagenarian* or sexagenarian* or dottering or decrepit or tottering or overaged or "oldest old" or supercentenarian*).mp. 3940510

2 exp Caregivers/ or (Carer* or caretak* or care tak* or care-tak* or caregiv* or care-giv*OR care giv* or carepartner* or care-partner* or care partner*).mp. 152451

3 exp Health Personnel/ or (acupuncturist* or allergist* or anatomist* or an?esthesiologist* or anesthetist* or audiologist* or cardiologist* or chiropractor* or clinician* or dentist* or dermatologist* or diabetologist* or dietician* or doctor* or doula or doulas or endocrinologist* or gastroenterologist* or general practitioner* or geriatrician* or gynecologist* or h?ematologist* or ((health or healthcare or hospital or medical) adj2 (worker* or workforce or personnel or practitioner* or provider* or professional* or employee* or staff* or navigator*)) or hospitalist* or immunologist* or intensivist* or internist* or medical resident* or midwife or midwives or neonatologist* or nephrologist* or neurologist* or neurosurgeon* or nurse or nurses or nutritionist* or obstetrician* or oncologist* or ophthalmologist* or optometrist* or osteopath or osteopaths or otolaryngologist* or pathologist* or p?ediatrician* or pharmacist* or pharmacologist* or phlebotomist* or physician* or podiatrist* or prosthetist* or psychologist* or psychiatrist* or pulmonologist* or radiographer* or radiologist* or radiotherapist* or rheumatologist* or surgeon* or therapist* or toxicologist* or urologist* or veterinarian*).mp. 2791924

4 exp Allied Health Personnel/ or Doulas/ or (allied health profession* or allied health* personnel or allied health* staff or allied health* practitioner* or allied health* employee* or allied health* worker* or athletic trainer* or audiologist* or community health worker* or counsellor* or counselor* or cytogenetic technologist* or dental auxiliaries or dental auxiliary or dental assistant* or dental hygienist* or dental technician* or denturist* or diagnostic molecular scientist* or dietitian* or doula or doulas or emergency medical technician* or EMT or EMTs or exercise physiologist* or family therapist* or health educator* or health information technologist* or healthcare assistant* or healthcare support worker* or histotechnologist* or home health* aide* or kinesiologist* or kinesiotherapist* or lactation consultant* or licensed practical nurse* or LPN or LPNs or medical dosimetrist* or medical laboratory scientist* or medical physicist* or medical records administrator* or medical receptionist* or medical secretar* or music therapist* or midwife* or nurse* aide* or nuclear medicine technologist* or nutritionist* or occupational therapist* or operating room technician* or ophthalmic assistant* or paramedic* or pathologist* assistant* or p?ediatric assistant* or pharmacy technician* or physical therapist* or physical therapy assistant* or physician assistant* or physiotherapist* or population program specialist* or prosthetist* or prosthet* technician* or psychiatric aide* or radiation therapist* or radiographer* or respiratory therapist* or sonographer* or (speech adj2 pathologist*)).mp. 228121

5 exp Health Communication/ or exp Health Education/ or (information-sharing or information sharing or information-exchange or "information exchange" or "knowledge transfer" or "data sharing" or "health communication" or "patient education" or "interpersonal communication").mp. 326220

6 1 and 2 42247

7 3 or 4 2867482

8 5 and 6 and 7 1689

**APA PsycInfo <1987 to April 2025 Week 4>**

1 exp Geriatrics/ or exp Aged/ or Health Services for the Aged/ or Senior Centers/ or (elders or elderly or geriatric* or gerontolog* or "old age" or "senior citizen*" or (seniors not "high school") or ((older or mature) adj3 (adult* or person* or people or patient or patients or man or men or woman or women)) or centenarian* or nonagenarian* or octogenarian* or septuagenarian* or sexagenarian* or dottering or decrepit or tottering or overaged or "oldest old" or supercentenarian*).mp. 195349

2 exp Caregivers/ or (Carer* or caretak* or care tak* or care-tak* or caregiv* or care-giv*OR care giv* or carepartner* or care-partner* or care partner*).mp. 101329

3 exp Health Personnel/ or (acupuncturist* or allergist* or anatomist* or an?esthesiologist* or anesthetist* or audiologist* or cardiologist* or chiropractor* or clinician* or dentist* or dermatologist* or diabetologist* or dietician* or doctor* or doula or doulas or endocrinologist* or gastroenterologist* or general practitioner* or geriatrician* or gynecologist* or h?ematologist* or ((health or healthcare or hospital or medical) adj2 (worker* or workforce or personnel or practitioner* or provider* or professional* or employee* or staff* or navigator*)) or hospitalist* or immunologist* or intensivist* or internist* or medical resident* or midwife or midwives or neonatologist* or nephrologist* or neurologist* or neurosurgeon* or nurse or nurses or nutritionist* or obstetrician* or oncologist* or ophthalmologist* or optometrist* or osteopath or osteopaths or otolaryngologist* or pathologist* or p?ediatrician* or pharmacist* or pharmacologist* or phlebotomist* or physician* or podiatrist* or prosthetist* or psychologist* or psychiatrist* or pulmonologist* or radiographer* or radiologist* or radiotherapist* or rheumatologist* or surgeon* or therapist* or toxicologist* or urologist* or veterinarian*).mp. 643872

4 exp Allied Health Personnel/ or Doulas/ or (allied health profession* or allied health* personnel or allied health* staff or allied health* practitioner* or allied health* employee* or allied health* worker* or athletic trainer* or audiologist* or community health worker* or counsellor* or counselor* or cytogenetic technologist* or dental auxiliaries or dental auxiliary or dental assistant* or dental hygienist* or dental technician* or denturist* or diagnostic molecular scientist* or dietitian* or doula or doulas or emergency medical technician* or EMT or EMTs or exercise physiologist* or family therapist* or health educator* or health information technologist* or healthcare assistant* or healthcare support worker* or histotechnologist* or home health* aide* or kinesiologist* or kinesiotherapist* or lactation consultant* or licensed practical nurse* or LPN or LPNs or medical dosimetrist* or medical laboratory scientist* or medical physicist* or medical records administrator* or medical receptionist* or medical secretar* or music therapist* or midwife* or nurse* aide* or nuclear medicine technologist* or nutritionist* or occupational therapist* or operating room technician* or ophthalmic assistant* or paramedic* or pathologist* assistant* or p?ediatric assistant* or pharmacy technician* or physical therapist* or physical therapy assistant* or physician assistant* or physiotherapist* or population program specialist* or prosthetist* or prosthet* technician* or psychiatric aide* or radiation therapist* or radiographer* or respiratory therapist* or sonographer* or (speech adj2 pathologist*)).mp. 78496

5 exp Health Communication/ or exp Health Education/ or (information-sharing or information sharing or information-exchange or "information exchange" or "knowledge transfer" or "data sharing" or "health communication" or "patient education" or "interpersonal communication").mp. 62426

6 1 and 2 14672

7 3 or 4 664584

8 5 and 6 and 7 214

**Embase <1988 to 2025 Week 18>**

1 exp geriatrics/ or aged/ or aged hospital patient/ or exp elderly care/ or frail elderly/ or gerontology/ or institutionalized elderly/ or very elderly/ or ("aging in place" or elders or elderly or geriatric* or gerodontic* or gerontol* or "old age" or "senior citizen*" or (seniors not "high school") or (older adj3 (adult* or person* or people or man or men or woman or women)) or centenarian* or nonagenarian* or octogenarian* or septuagenarian* or sexagenarian* or dottering or decrepit or tottering or overaged or "oldest old" or supercentenarian*).mp. 4192801

2 exp Caregivers/ or (Carer* or caretak* or care tak* or care-tak* or caregiv* or care-giv*OR care giv* or carepartner* or care-partner* or care partner*).mp. 221505

3 exp health care personnel/ or (acupuncturist* or allergist* or anatomist* or an?esthesiologist* or anesthetist* or audiologist* or cardiologist* or chiropractor* or clinician* or dentist* or dermatologist* or diabetologist* or dietician* or doctor* or doula or doulas or endocrinologist* or gastroenterologist* or general practitioner* or geriatrician* or gynecologist* or h?ematologist* or ((health or healthcare or hospital or medical) adj2 (worker* or workforce or personnel or practitioner* or provider* or professional* or employee* or staff* or navigator*)) or hospitalist* or immunologist* or intensivist* or internist* or medical resident* or midwife or midwives or neonatologist* or nephrologist* or neurologist* or neurosurgeon* or nurse or nurses or nutritionist* or obstetrician* or oncologist* or ophthalmologist* or optometrist* or osteopath or osteopaths or otolaryngologist* or pathologist* or p?ediatrician* or pharmacist* or pharmacologist* or phlebotomist* or physician* or podiatrist* or prosthetist* or psychologist* or psychiatrist* or pulmonologist* or radiographer* or radiologist* or radiotherapist* or rheumatologist* or surgeon* or therapist* or toxicologist* or urologist* or veterinarian*).mp. 3930446

4 exp paramedical personnel/ or doula/ or (allied health profession* or allied health* personnel or allied health* staff or allied health* practitioner* or allied health* employee* or allied health* worker* or athletic trainer* or audiologist* or community health worker* or counsellor* or counselor* or cytogenetic technologist* or dental auxiliaries or dental auxiliary or dental assistant* or dental hygienist* or dental technician* or denturist* or diagnostic molecular scientist* or dietitian* or doula or doulas or emergency medical technician* or EMT or EMTs or exercise physiologist* or family therapist* or health educator* or health information technologist* or healthcare assistant* or healthcare support worker* or histotechnologist* or home health* aide* or kinesiologist* or kinesiotherapist* or lactation consultant* or licensed practical nurse* or LPN or LPNs or medical dosimetrist* or medical laboratory scientist* or medical physicist* or medical records administrator* or medical receptionist* or medical secretar* or music therapist* or midwife* or nurse* aide* or nuclear medicine technologist* or nutritionist* or occupational therapist* or operating room technician* or ophthalmic assistant* or paramedic* or pathologist* assistant* or p?ediatric assistant* or pharmacy technician* or physical therapist* or physical therapy assistant* or physician assistant* or physiotherapist* or population program specialist* or prosthetist* or prosthet* technician* or psychiatric aide* or radiation therapist* or radiographer* or respiratory therapist* or sonographer* or (speech adj2 pathologist*)).mp. 773783

5 exp Health Communication/ or exp Health Education/ or (information-sharing or information sharing or information-exchange or "information exchange" or "knowledge transfer" or "data sharing" or "health communication" or "patient education" or "interpersonal communication").mp. 668878

6 1 and 2 54234

7 3 or 4 4011534

8 5 and 6 and 7 3206

**CINAHL Plus Full Text**

| **#** | **Query** | **Results** |
| --- | --- | --- |
| S1 | (MH "Geriatrics") or (MH "Aged, Hospitalized") or (MH "Aged+") or (MH "Senior Centers") or (MH "Gerontologic Care") or (MH "Geriatricians") OR (MH "Gerontologic Nursing+") OR (MH "Health Services for the Aged") or ("aging in place" or elders or elderly or geriatric* or gerontolog* or gerodontic* or "old age*" or (seniors not "high school") or "senior citizen*" or (older N3 (patient* or adult* or person* or people or man or men or woman or women)) or centenarian* or nonagenarian* or octogenarian* or septuagenarian* or sexagenarian* or dottering or decrepit or tottering or overaged or "oldest old") | 1,118,331 |
| S2 | (MH “Caregivers”) or (Carer* OR caretak* OR care tak* OR care-tak* OR caregiv* OR care-giv*OR care giv* OR carepartner* OR care-partner* OR care partner*) | 225,970 |
| S3 | (MH "Health Personnel+") or (acupuncturist* or allergist* or anatomist* or an#esthesiologist* or anesthetist* or audiologist* or cardiologist* or chiropractor* or clinician* or dentist* or dermatologist* or diabetologist* or dietician* or doctor* or doula or doulas or endocrinologist* or gastroenterologist* or "general practitioner*" or geriatrician* or gynecologist* or h#ematologist* or ((health or healthcare or hospital or medical) N2 (worker* or workforce or personnel or practitioner* or provider* or professional* or employee* or staff* or navigator*)) or hospitalist* or immunologist* or intensivist* or internist* or "medical resident*" or midwife or midwives or neonatologist* or nephrologist* or neurologist* or neurosurgeon* or nurse or nurses or nutritionist* or obstetrician* or oncologist* or ophthalmologist* or optometrist* or osteopath or osteopaths or otolaryngologist* or pathologist* or p#ediatrician* or pharmacist* or pharmacologist* or phlebotomist* or physician* or podiatrist* or prosthetist* or psychologist* or psychiatrist* or pulmonologist* or radiographer* or radiologist* or radiotherapist* or rheumatologist* or surgeon* or therapist* or toxicologist* or urologist* or veterinarian*) | 1,611,033 |
| S4 | (MH “Allied Health Personnel”) or (MH “Doulas”) or (allied health profession* or allied health* personnel or allied health* staff or allied health* practitioner* or allied health* employee* or allied health* worker* or athletic trainer* or audiologist* or community health worker* or counsellor* or counselor* or cytogenetic technologist* or dental auxiliaries or dental auxiliary or dental assistant* or dental hygienist* or dental technician* or denturist* or diagnostic molecular scientist* or dietitian* or doula or doulas or emergency medical technician* or EMT or EMTs or exercise physiologist* or family therapist* or health educator* or health information technologist* or healthcare assistant* or healthcare support worker* or histotechnologist* or home health* aide* or kinesiologist* or kinesiotherapist* or lactation consultant* or licensed practical nurse* or LPN or LPNs or medical dosimetrist* or medical laboratory scientist* or medical physicist* or medical records administrator* or medical receptionist* or medical secretar* or music therapist* or midwife* or nurse* aide* or nuclear medicine technologist* or nutritionist* or occupational therapist* or operating room technician* or ophthalmic assistant* or paramedic* or pathologist* assistant* or p#ediatric assistant* or pharmacy technician* or physical therapist* or physical therapy assistant* or physician assistant* or physiotherapist* or population program specialist* or prosthetist* or prosthet* technician* or psychiatric aide* or radiation therapist* or radiographer* or respiratory therapist* or sonographer* or (speech adj2 pathologist*)) | 257,379 |
| S5 | (MH “Health Communication”) or (MH “Health Education”) or (information-sharing OR information sharing or information-exchange or "information exchange" or "knowledge transfer" or "data sharing" or "health communication" or "patient education" or "interpersonal communication") | 131,682 |
| S6 | S1 AND S2 | 53,471 |
| S7 | S3 OR S4 | 1,660,629 |
| S8 | S5 AND S6 AND S7 | 1,347 |

**Cochrane CENTRAL**

Date Run: 06/05/2025 05:35:21

ID Search Hits

#1 MeSH descriptor: [Geriatrics] explode all trees 297

#2 (("aging in place" or elders or elderly or geriatric* or gerodontic* or gerontol* or "old age" or "senior citizen*" or (seniors not "high school") or (older adj3 (adult* or person* or people or man or men or woman or women)) or centenarian* or nonagenarian* or octogenarian* or septuagenarian* or sexagenarian* or dottering or decrepit or tottering or overaged or "oldest old" or supercentenarian*)):ti,ab,kw (Word variations have been searched) 78599

#3 #1 or #2 78599

#4 MeSH descriptor: [Caregivers] explode all trees 4017

#5 (Carer* or caretak* or care tak* or care-tak* or caregiv* or care-giv*OR care giv* or carepartner* or care-partner* or care partner*):ti,ab,kw 62471

#6 #4 or #5 62471

#7 #3 and #6 4309

#8 ((acupuncturist* or allergist* or anatomist* or an?esthesiologist* or anesthetist* or audiologist* or cardiologist* or chiropractor* or clinician* or dentist* or dermatologist* or diabetologist* or dietician* or doctor* or doula or doulas or endocrinologist* or gastroenterologist* or general practitioner* or geriatrician* or gynecologist* or h?ematologist* or ((health or healthcare or hospital or medical) adj2 (worker* or workforce or personnel or practitioner* or provider* or professional* or employee* or staff* or navigator*)) or hospitalist* or immunologist* or intensivist* or internist* or medical resident* or midwife or midwives or neonatologist* or nephrologist* or neurologist* or neurosurgeon* or nurse or nurses or nutritionist* or obstetrician* or oncologist* or ophthalmologist* or optometrist* or osteopath or osteopaths or otolaryngologist* or pathologist* or p?ediatrician* or pharmacist* or pharmacologist* or phlebotomist* or physician* or podiatrist* or prosthetist* or psychologist* or psychiatrist* or pulmonologist* or radiographer* or radiologist* or radiotherapist* or rheumatologist* or surgeon* or therapist* or toxicologist* or urologist* or veterinarian*)):ti,ab,kw 224317

#9 ((allied health profession* or allied health* personnel or allied health* staff or allied health* practitioner* or allied health* employee* or allied health* worker* or athletic trainer* or audiologist* or community health worker* or counsellor* or counselor* or cytogenetic technologist* or dental auxiliaries or dental auxiliary or dental assistant* or dental hygienist* or dental technician* or denturist* or diagnostic molecular scientist* or dietitian* or doula or doulas or emergency medical technician* or EMT or EMTs or exercise physiologist* or family therapist* or health educator* or health information technologist* or healthcare assistant* or healthcare support worker* or histotechnologist* or home health* aide* or kinesiologist* or kinesiotherapist* or lactation consultant* or licensed practical nurse* or LPN or LPNs or medical dosimetrist* or medical laboratory scientist* or medical physicist* or medical records administrator* or medical receptionist* or medical secretar* or music therapist* or midwife* or nurse* aide* or nuclear medicine technologist* or nutritionist* or occupational therapist* or operating room technician* or ophthalmic assistant* or paramedic* or pathologist* assistant* or p?ediatric assistant* or pharmacy technician* or physical therapist* or physical therapy assistant* or physician assistant* or physiotherapist* or population program specialist* or prosthetist* or prosthet* technician* or psychiatric aide* or radiation therapist* or radiographer* or respiratory therapist* or sonographer* or (speech adj2 pathologist*))):ti,ab,kw 47320

#10 #8 or #9 247559

#11 MeSH descriptor: [Health Communication] explode all trees 388

#12 MeSH descriptor: [Health Education] explode all trees 26247

#13 ((information-sharing or information sharing or information-exchange or "information exchange" or "knowledge transfer" or "data sharing" or "health communication" or "patient education" or "interpersonal communication")):ti,ab,kw 21694

#14 #11 or #12 or #13 36673

#15 #7 and #10 and #14 123
